# Antioxidant activity of the thioredoxin system

**DOI:** 10.52601/bpr.2023.230002

**Published:** 2023-02-28

**Authors:** Zihua Liu

**Affiliations:** 1 Department of blood transfusion school of second hospital, Lanzhou University, Lanzhou 730030, China

**Keywords:** Thioredoxin, Thioredoxin reductase, Cancer, Neurodegenerative disease

## Abstract

The thioredoxin system is composed of thioredoxin (Trx), thioredoxin reductase (TR) and reduced nicotinamide adenine dinucleotide phosphate. Trx is an important antioxidant molecule that can resist cell death caused by various stresses and plays a prominent role in redox reactions. TR is a protein that contains selenium (selenocysteine), in three main forms, namely, TR1, TR2 and TR3. TR1, TR2 and TR3 are mainly distributed in the cytoplasm, mitochondria, and testes, respectively. TR can regulate cell growth and apoptosis. After a cell becomes cancerous, the expression of TR is increased to promote cell growth and metastasis. The Trx system is closely related to neurodegenerative diseases, parasitic infections, acquired immunodeficiency syndrome, rheumatoid arthritis, hypertension, myocarditis, and so on. In addition, the Trx system can remove the reactive oxygen species in the body and keep the inside and outside of the cell in a balanced state. In summary, the Trx system is an important target for the drug treatment of many diseases.

## INTRODUCTION OF THE THIOREDOXIN SYSTEM

**Figure 1:**
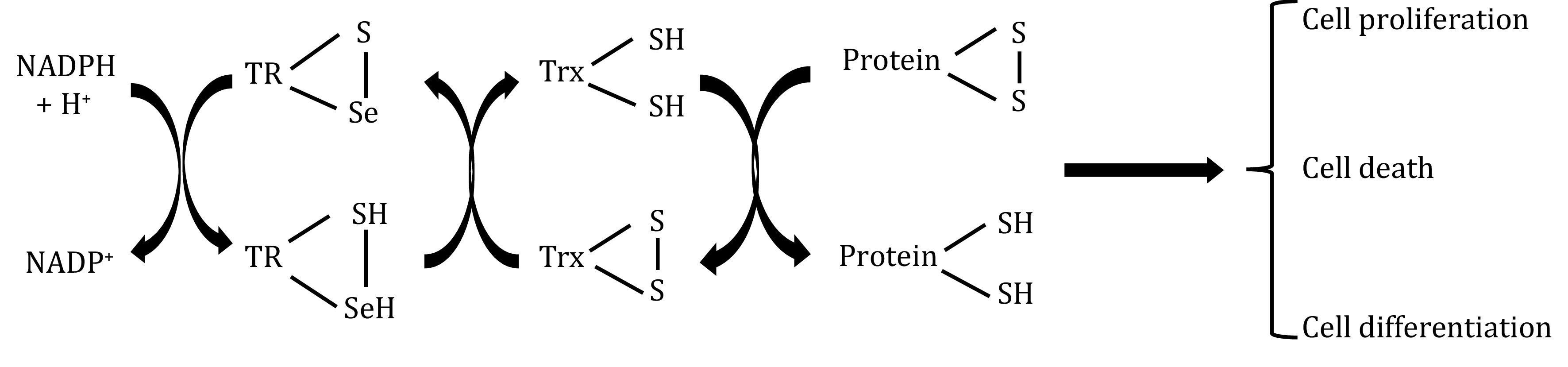
Diagram of the interaction of the Trx system with substrate molecules (Zhang *et al.* 2018)

The thioredoxin system is composed of thioredoxin (Trx), thioredoxin reductase (TR) and reduced nicotinamide adenine dinucleotide phosphate (NADPH). Trx and TR are present in many tumor patient tissue and blood samples. The catalytic process of the Trx system is shown in Fig. 1.

The p*K*a (−5.9) value of selenocysteine (SEC) is much lower than that of cysteine (−8.5). The Trx system is an NADPH-dependent reductase system that includes the Trx1 (cytoplasmic), Trx2 (mitochondrial), and Trx3 (testicular specific) subtypes (Powis and Kirkpatrick 2007; Roman *et al.* 2020; Xinastle-Castillo and Landa 2022). The selenoprotein TR is the only reducing agent of Trx and has three isoforms (TR1, TR2, and TR3) in mammals (Powis and Kirkpatrick 2007). The structure of TR2 is similar to that of TR1 as both contain a C-terminal SEC active region (Sun *et al.* 1999) and can enter mitochondria to produce mature proteins.

### Thioredoxin (Trx)

**Figure 2:**
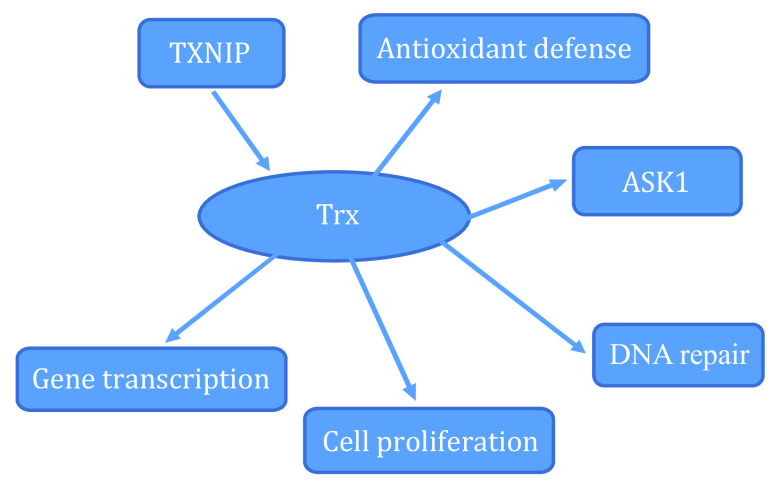
Function of Trx (Bjorklund *et al.* 2022; Liu and Min 2002; Maulik and Das 2008; Schenk *et al.* 1994; Shelar *et al.* 2015; Yi *et al.* 2019)

Trx is an important antioxidant molecule. Laurent *et al*. were the first to obtain Trx in *E. coli* in 1964. Trx1 can be combined with peroxidase and H_2_O_2_ to reduce oxidized proteins. Mice in which Trx1 and Trx2 are knocked out experience embryo death. Trx plays an outstanding role in redox reactions (Zhang *et al.* 2017). Trx was originally found to be used as electron donor for RNA reductase in *E. coli* (Laurent *et al.* 1964). The electron donor of RNA reductase is composed of α helix and β folded connections, and it is stably high in protein (Aguado-Llera *et al.* 2011; Gasdaska *et al.* 1994). Trx can be reduced by TR in the presence of NADPH to form oxygen in the oxidation state (Zhang *et al.* 2020). Trx, acting as a growth factor, interacts with TXNIP to perform the following roles: (1) regulating the immune function (Bjorklund *et al.* 2022); (2) scavenging intracellular reactive oxygen species (ROS), regulating other enzyme activities and reducing oxidative stress; (3) maintaining normal intracellular redox homeostasis (Zhang *et al.* 2020) and reducing Gpx3 (Bjornstedt *et al.* 1994)_;_ (4) scavenging singlet oxygen and hydroxyl radicals through ROS (Yi *et al.* 2019) and regulating p53 and Nrf2 gene expression (Maulik and Das 2008); (5) regulating apoptotic protein expression through the nuclear factor kappa B (NF-κB) signaling pathway (Schenk *et al.* 1994); and (6) regulating apoptosis through ASK1 (Liu and Min 2002). The function of Trx is shown in Fig. 2.

### Thioredoxin reductase (TR)

TR is a selenium enzyme that is closely related to lung cancer. TR can remove ROS *in vivo* and balance cells and tissues in the inner and lateral positions. After cell canceration, the expression of TR1 is increased, promoting cell growth and metastasis.

#### Structure and type of TR

Macromolecule TR mainly exists in higher eukaryotes, with a molecular weight of 55 kDa (Williams *et al.* 2000). TRl, TR2 and TR3 are mainly distributed in the cytoplasm, mitochondria and testes, respectively (Liu *et al.* 2023). Flavin adenine exists in TR1 and TR2 dinucleotide (FAD) binding and action sites, and TR3 contains the CVNVGC active site region. The end contains a relatively conserved active site, namely, Cys-Val-Asn-Val-GLy-Cys, which has a redox catalytic active site. The C-terminus contains Gly-Cys-Sec-Gly, and the binding domain of NADPH and FAD. They are active sites. The two regions are connected by a folded chain; the action site changes under catalytic conditions, the binding domain rotates 66° from NADPH to FAD, and the substrate is exposed (Lennon *et al.* 1999).

#### Biological function of TR

SEC is present in TR (Tuladhar *et al.* 2019), which is an important site for its enzyme activity (Zhong *et al.* 2000). TR can act on oxidized Trx to reduce Trx (Zhong *et al.* 2000), and NADPH is oxidized to NADP^+ ^ (Zhang *et al.* 2018). After Trx is reduced, the disulfide bonds of other proteins are reduced, thus exerting biological activity. In addition, TR can also reduce antioxidants, protein disulfide isomerase (PDI) (Che *et al.* 2020; Ogata *et al.* 2022), lipid peroxides and H_2_O_2_ (Ghneim *et al.* 2022). TR plays an antioxidant role through the catalytic regeneration of coenzyme Q10 (Preci *et al.* 2021). TR1 is present in tissue cytoplasm. The human TR gene is located on chromosomes 12Q23–Q24.1 (Gasdaska *et al.* 1996) and contains 3826 bp base cDNA (Gasdaska *et al.* 1995) encoding a total of 497 amino acid sequences (Jan *et al.* 2014).

#### Catalytic mechanism of TR

The SEC insertion sequence is required when SEC binds to the polypeptide chain of selenoprotein. The combined action of the selenocysteine insertion sequence (SECIS) element, SECIS binding protein 2 (SBP2), and other related compounds in the cell is needed. The binding of SEC greatly affects the activity of TR (Papp *et al.* 2007), and insufficient synthesis of SEC components changes TR storage (Lu *et al.* 2009). TR has unique properties in mammals (Liu and Min 2002), while TR is a small endogenous dimer flavin protein (molecular weight: approximately 33.9 kDa) in bacteria (Kreimer *et al.* 1997).

#### TR regulates cell growth and apoptosis

**Figure 3:**
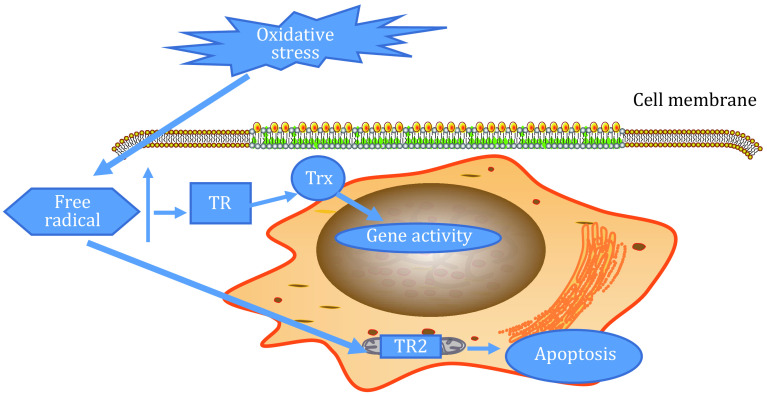
Function of the TR system (Miranda-Vizuete *et al.* 2000; Nguyen *et al.* 2006)

Enzyme inactivation of TR1 can lead to early embryo death (Jakupoglu *et al.* 2005), and this suggests that TRl is an extremely important substance in embryonic development. Nrf2 can regulate TR1 and affect cell proliferation (Gao *et al.* 2020), and TR is the only reducing agent that can reduce Trx. Therefore, through Trx or its own specific substrate, TR can play its redox role. The function of the TR system is shown in Fig. 3.

### Relationship between the thioredoxin system and diseases

There are many diseases related to the Trx system, including tumors, acquired immunodeficiency syndrome (AIDS), parasitic infections, rheumatoid arthritis (RA), hypertension, myocarditis and neurodegenerative diseases.

#### Acquired immunodeficiency syndrome (AIDS)

TR levels are high in cancer cells (Gencheva and Arner 2022). The expression of TR is increased in leukemia, solid tumors and lymphatic carcinoma (Campbell *et al.* 2021). At the early stages of a tumor, Trx protects against cancer. Trx was found to promote the expression of VEGF (Welsh *et al.* 2002). The development of AIDS is related to oxidative damage in the body. The synthesis of the endogenous antioxidant selenoprotein TR1 is decreased, and glutathione peroxidase and small-molecule selenium compounds are increased.

#### Parasitic infections and rheumatoid arthritis (RA)

A study found that *Plasmodium falciparum* contains TR1 (Krnajski *et al.* 2002). TR and its related Trx and glutenin were also found in *Schistosoma japonicum*. The original enzymes (Sun *et al.* 2001) Trx and TR are significantly increased in the synovial fluid and tissues of RA patients, but not in plasma (Maurice *et al.* 1999). The gold-containing compounds aurioglucosamine and ranonophen are clinically used to treat RA. They are highly effective inhibitors of TR (Smith *et al.* 1999).

#### Hypertension and myocarditis

Angiotensin Ⅱ can increase the activity of NADPH oxidase, cause mitochondrial function damage, produce ROS, and cause hyperemia and pressure disease (de Cavanagh *et al.* 2009). TR2 mainly exists in mitochondria. Matsushima found that overexpression of TR2 results in good prognosis after myocardial injury in rats (Matsushima *et al.* 2006). In transgenic rats with high expression of TR2, ROS production and the expression of NADPH oxidase decrease. TR2 can maintain the normal function of endothelial cells (Kameritsch *et al.* 2021). Moreover, TR gene silencing in the heart increases the probability of oxidative stress and myocardial hypertrophy (Li *et al.* 2017). The TR activity in the myocardium of pigs fed with low selenium decreases (Sun *et al.* 2020). The expression of Trx in myocarditis is increased, and motiflrin can reduce myocardial injury (Yuan *et al.* 2003).

#### Neurodegenerative diseases

The human brain needs oxygen, oxidative polyunsaturated fatty acids and cholesterol. It is more susceptible to free radical damage than other tissues are, and oxidative damage is likely to occur with increasing age. Therefore, oxidative damage is considered to be a major factor in neurodegenerative diseases in the elderly population (Singh *et al.* 2019). The Trx system is abundant in nerve cells and axons. Trx and TR are mainly located in the cytoplasm and result in oxidative damage. They are closely associated with neurodegenerative diseases. Oxidative damage is associated with damage to the endoplasmic reticulum and mitochondria, which can induce neurosis, apoptosis and protein misfolding. The level of Trxl in the brains of AD patients is low, and P-amyloid peptide accumulates in large quantities (Lovell *et al.* 2000). Meanwhile, overexpression of amyloid peptides can reduce the expression and activity of TR, thus reducing the ability of Trxl to reduce substrates (Lamoke *et al.* 2012). These studies suggest that the loss of Trx or TR function is likely to result in neuronal decline (Seyfried and Wullner 2007).

**Figure 4:**
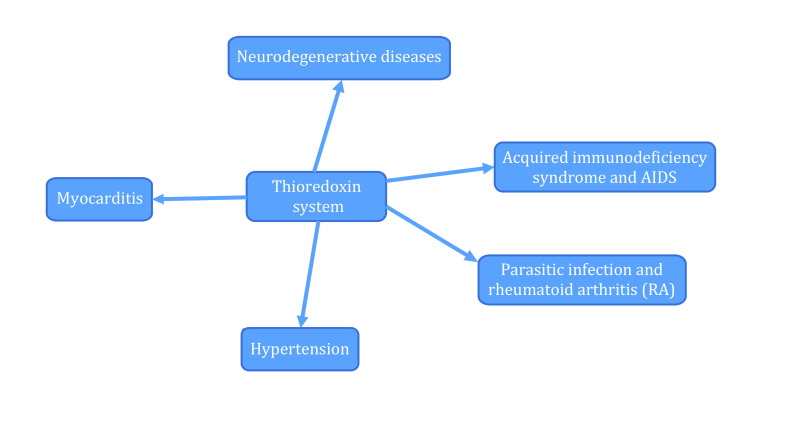
Relationship between the Trx system and diseases (Kudin *et al*. 2012; Lewin *et al*. 2001)

Alzheimer's disease, Parkinson's disease and Huntington's disease are the major neurodegenerative diseases. The human brain requires oxygen and is prone to oxidative damage, which is likely to occur with age. Therefore, oxidative damage is considered to be the main factor for the occurrence of neurodegenerative diseases in the elderly (Maiuri *et al.* 2019). Trx and TR are mainly located in the cytoplasm and closely associated with neurodegenerative diseases. Oxidative damage is related to damage to the endoplasmic reticulum and mitochondria, and induces neuronal apoptosis and protein misfolding. The level of Trx1 in the brains of patients with Alzheimer's disease is low, and amyloid β protein (Aβ) accumulates massively (Lovell *et al.* 2000). Overexpression of Trx1 protects cells against cytotoxicity induced by Aβ. Animal experiments have shown that overexpression of Aβ reduces the expression and activity of TR, resulting in the lack of substrate reduction ability of Trx1 (Lamoke *et al.* 2012). The results of previous studies indicate that the loss of Trx or TR function is likely to cause a decline in neuronal bias (Seyfried and Wullner 2007), but the specific mechanism is still unclear. It has been reported that TR and Trx exert protective effects against neurotoxicity induced by Aβ (Lovell *et al.* 2000). Retinal neurotoxicity mediated by Aβ includes damage to the Trx system and reduction in TR activity (Lamoke *et al.* 2012). In addition, Kudin *et al*. (Kudin *et al.* 2012) confirmed that the Trx2 system plays an important role in H_2_O_2_ detoxification in the hippocampus of rats. These findings highlight TR as an important source of reductive energy in the brain. TR can maintain the intracellular reduction state, and remove lipid peroxides and H_2_O_2_ under the action of NADPH (Lewin *et al.* 2001). Lovell *et al*. (Lovell *et al.* 2000) co-cultured Aβ with hippocampal nerve cells and found that the survival rate of nerve cells is low when Aβ is co-cultured with hippocampal nerve cells for 12 h. However, the survival rates of Aβ, TR and hippocampal neurons increased after co-culturing for 12 h, suggesting that TR has a protective effect on Aβ-induced neurocytotoxicity. The relationship between the Trx system and diseases is shown in Fig. 4.

## SEVERAL COMMONLY USED INHIBITORS OF TR

**Figure 5:**
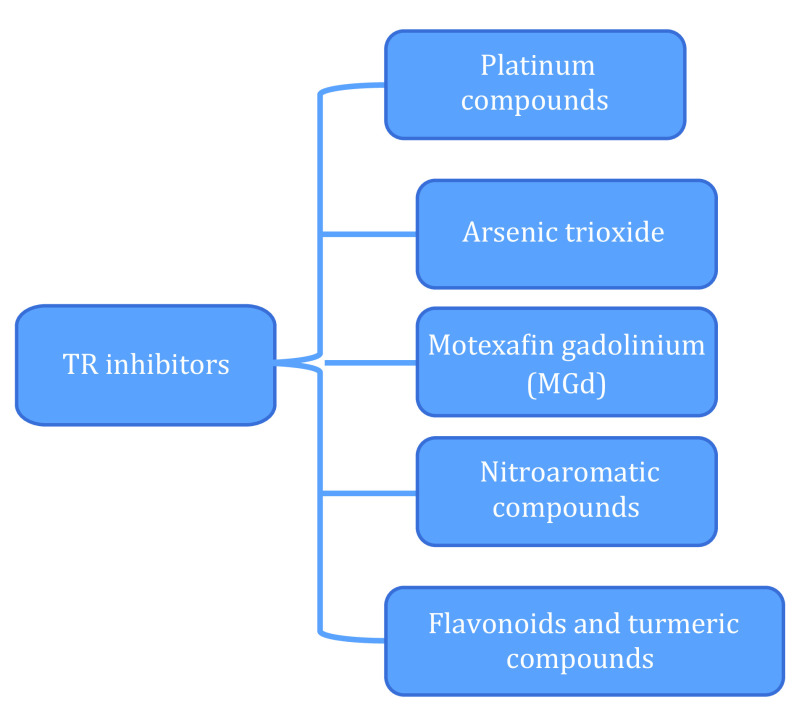
Inhibitors of TR (Eberle *et al.* 2018; Hashemy *et al.* 2006; Liu *et al.* 2009; Marzano *et al.* 2007; Park 2020)

Over the last decade, many inhibitors have been developed that target the Trx system. Herein, several commonly used TR inhibitors are listed. (1) Platinum compounds include oxaliplatin, cisplatin, carboplatin and ginovine (Marzano *et al.* 2007). (2) Arsenic trioxide is an irreversible inhibitor of TR (Park 2020). (3) Motexafin gadolinium (MGd) is a porphyrin compound whose target molecule is TR1 in the cytoplasm (Hashemy *et al.* 2006). MGd can block the reducing activity of TR1 and generate peroxide (Liu *et al.* 2009). (4) Nitroaromatic compounds. the most typical of which is 1-chlorophyll-2,4-dinitrobenzene, are also widely used inhibitors for TR. (5) Flavonoids and turmeric compounds, which have long been used in tumor therapy, are mediated by the TR system. Turmeric compounds can inhibit TR. Flavonoids have an inhibitory effect on thioredoxin A1 of *Corynebacterium pseudotuberculosis* (Eberle *et al.* 2018). The inhibitors of TR are shown in Fig. 5.

## CONCLUSION

In summary, the Trx system is an important antioxidant reduction system *in vivo* that is closely related to cell proliferation, differentiation and death, and is associated with tumors, neurodegenerative diseases, rheumatoid arthritis, hypertension, myocarditis and other diseases. The Trx system can remove excessive ROS generated in the body and keep the cell in balance. Intervention of the Trx system is an important target of drugs.

## Conflict of interest

Zihua Liu declare that they have no conflict of interest.

## References

[bAguadoLlera2011] (2011). The conformational stability and biophysical properties of the eukaryotic thioredoxins of *Pisum sativum* are not family-conserved. PLoS One.

[bBjorklund2022] (2022). The role of the thioredoxin system in brain diseases. Antioxidants (Basel).

[bBjornstedt1994] (1994). The thioredoxin and glutaredoxin systems are efficient electron donors to human plasma glutathione peroxidase. J Biol Chem.

[bCampbell2021] (2021). Engineered resveratrol-loaded fibrous scaffolds promotes functional cardiac repair and regeneration through Thioredoxin-1 mediated VEGF pathway. Int J Pharm.

[bChe2020] (2020). Thioredoxin and protein-disulfide isomerase selectivity for redox regulation of proteins in *Corynebacterium glutamicum*. J Gen Appl Microbiol.

[bde2009] (2009). Angiotensin II, mitochondria, cytoskeletal, and extracellular matrix connections: an integrating viewpoint. Am J Physiol-Heart C.

[bEberle2018] (2018). Inhibition of thioredoxin A1 from Corynebacterium pseudotuberculosis by polyanions and flavonoids. Int J Biol Macromol.

[bGao2020] (2020). SLC27A5 deficiency activates NRF2/TXNRD1 pathway by increased lipid peroxidation in HCC. Cell Death Differ.

[bGasdaska1996] Gasdaska JR, Gasdaska PY, Gallegos A, Powis G (1996) Human thioredoxin reductase gene localization to chromosomal position 12q23-q24.1 and mRNA distribution in human tissue. Genomics 37(2): 257-259

[bGasdaska1995] (1995). Cloning and sequencing of a human thioredoxin reductase. FEBS Lett.

[bGasdaska1994] (1994). The predicted amino acid sequence of human thioredoxin is identical to that of the autocrine growth factor human adult T-cell derived factor (ADF): thioredoxin mRNA is elevated in some human tumors. Biochim Biophys Acta..

[bGencheva2022] (2022). Thioredoxin reductase inhibition for cancer therapy. Annu Rev Pharmacol Toxicol.

[bGhneim2022] (2022). Comprehensive investigations of key mitochondrial metabolic changes in senescent human fibroblasts. Korean J Physiol Pharmacol.

[bHashemy2006] (2006). Motexafin gadolinium, a tumor-selective drug targeting thioredoxin reductase and ribonucleotide reductase. J Biol Chem.

[bJakupoglu2005] (2005). Cytoplasmic thioredoxin reductase is essential for embryogenesis but dispensable for cardiac development. Mol Cell Biol.

[bJan2014] (2014). Acetaminophen reactive intermediates target hepatic thioredoxin reductase. Chem Res Toxicol.

[bKameritsch2021] (2021). The mitochondrial thioredoxin reductase system (TrxR2) in vascular endothelium controls peroxynitrite levels and tissue integrity. Proc Natl Acad Sci USA.

[bKreimer1997] (1997). Two closely linked genes encoding thioredoxin and thioredoxin reductase in *Clostridium litorale*. Arch Microbiol.

[bKrnajski2002] (2002). Thioredoxin reductase is essential for the survival of *Plasmodium falciparum* erythrocytic stages. J Biol Chem.

[bKudin2012] (2012). The contribution of thioredoxin-2 reductase and glutathione peroxidase to H_2_O_2_ detoxification of rat brain mitochondria. BBA-Bioenergetics.

[bLamoke2012] (2012). Loss of thioredoxin function in retinas of mice overexpressing amyloid β. Free Radical Bio Med.

[bLaurent1964] (1964). Enzymatic synthesis of deoxyribonucleotides. IV. Isolation and characterization of thioredoxin, the hydrogen donor from Escherichia coli B. J Biol Chem.

[bLennon1999] (1999). Crystal structure of reduced thioredoxin reductase from *Escherichia coli*: structural flexibility in the isoalloxazine ring of the flavin adenine dinucleotide cofactor. Protein Sci.

[bLewin2001] (2001). Thioredoxin reductase and cytoplasmic glutathione peroxidase activity in human foetal and neonatal liver. BBA-GEN Subjects.

[bLi2017] (2017). Thioredoxin 2 offers protection against mitochondrial oxidative stress in H9c2 cells and against myocardial hypertrophy induced by hyperglycemia. Int J Mol Sci.

[bLiu2023] (2023). Gpx4, Selenov, and Txnrd3 are three most testis-abundant selenogenes resistant to dietary selenium concentrations and actively expressed during reproductive ages in rats. Biol Trace Elem Res.

[bLiu2002] (2002). Thioredoxin promotes ASK1 ubiquitination and degradation to inhibit ASK1-mediated apoptosis in a redox activity-independent manner. Circ Res.

[bLiu2009] (2009). Inhibition of thioredoxin reductase by mansonone F analogues: implications for anticancer activity. Chem-Biol Interact.

[bLovell2000] (2000). Decreased thioredoxin and increased thioredoxin reductase levels in Alzheimer's disease brain. Free Radic Biol Med.

[bLu2009] (2009). Penultimate selenocysteine residue replaced by cysteine in thioredoxin reductase from selenium-deficient rat liver. FASEB J.

[bMaiuri2019] (2019). DNA repair signaling of huntingtin: the next link between late-onset neurodegenerative disease and oxidative DNA damage. DNA Cell Biol.

[bMarzano2007] (2007). Inhibition of thioredoxin reductase by auranofin induces apoptosis in cisplatin-resistant human ovarian cancer cells. Free Radical Bio Med.

[bMatsushima2006] (2006). Overexpression of mitochondrial peroxiredoxin-3 prevents left ventricular remodeling and failure after myocardial infarction in mice. Circulation.

[bMaulik2008] (2008). Emerging potential of thioredoxin and thioredoxin interacting proteins in various disease conditions. Biochim Biophys Acta..

[bMaurice1999] (1999). Expression of the thioredoxin-thioredoxin reductase system in the inflamed joints of patients with rheumatoid arthritis. Arthritis Rheum.

[bMirandaVizuete2000] (2000). The mitochondrial thioredoxin system. Antioxid Redox Signal.

[bNguyen2006] (2006). Thioredoxin reductase as a novel molecular target for cancer therapy. Cancer Lett.

[bOgata2022] (2022). Thiol-based antioxidants and the epithelial/mesenchymal transition in cancer. Antioxid Redox Signal.

[bPapp2007] (2007). From selenium to selenoproteins: synthesis, identity, and their role in human health. Antioxid Redox Signal.

[bPark2020] (2020). Upregulation of thioredoxin and its reductase attenuates arsenic trioxide-induced growth suppression in human pulmonary artery smooth muscle cells by reducing oxidative stress. Oncol Rep.

[bPowis2007] (2007). Thioredoxin signaling as a target for cancer therapy. Curr Opin Pharmacol.

[bPreci2021] (2021). Oxidative damage and antioxidants in cervical cancer. Int J Gynecol Cancer.

[bRoman2020] Roman MG, Flores LC, Cunningham GM, Cheng C, Allen C, Hubbard GB, Bai Y, Saunders TL, Ikeno Y (2020) Thioredoxin and aging: what have we learned from the survival studies? Aging Pathobiol Ther 2(3): 126-133

[bSchenk1994] (1994). Distinct effects of thioredoxin and antioxidants on the activation of transcription factors NF-B and AP-1. Proc Natl Acad Sci USA.

[bSeyfried2007] (2007). Inhibition of thioredoxin reductase induces apoptosis in neuronal cell lines: Role of glutathione and the MKK4/JNK pathway. Biochem Bioph Res Commun.

[bShelar2015] (2015). Thioredoxin-dependent regulation of AIF-mediated DNA damage. Free Radic Biol Med.

[bSingh2019] (2019). Oxidative stress: a key modulator in neurodegenerative diseases. Molecules.

[bSmith1999] (1999). Aurothioglucose inhibits murine thioredoxin reductase activity *in vivo*. J Nutr.

[bSun2001] (2001). Selenoprotein oxidoreductase with specificity for thioredoxin and glutathione systems. Proc Natl Acad Sci USA.

[bSun1999] (1999). Redox regulation of cell signaling by selenocysteine in mammalian thioredoxin reductases. J Biol Chem.

[bSun2020] (2020). Selenium supplementation protects against oxidative stress-induced cardiomyocyte cell cycle arrest through activation of PI3K/AKT. Metallomics.

[bTuladhar2019] (2019). Effectors of thioredoxin reductase: brevetoxins and manumycin — A. Comp Biochem Physiol C Toxicol Pharmacol.

[bWelsh2002] (2002). The redox protein thioredoxin-1 (Trx-1) increases hypoxia-inducible factor 1 alpha protein expression: Trx-1 overexpression results in increased vascular endothelial growth factor production and enhanced tumor angiogenesis. Cancer Res.

[bWilliams2000] (2000). Thioredoxin reductase — Two modes of catalysis have evolved. Eur J Biochem.

[bXinastleCastillo2022] (2022). Physiological and modulatory role of thioredoxins in the cellular function. Open Med.

[bYi2019] (2019). Glutathione peroxidase 3 (GPX3) suppresses the growth of melanoma cells through reactive oxygen species (ROS)-dependent stabilization of hypoxia-inducible factor 1-α and 2-α. J Cell Biochem.

[bYuan2003] (2003). Temocapril treatment ameliorates autoimmune myocarditis associated with enhanced cardiomyocyte thioredoxin expression. Mol Cell Biochem.

[bZhang2018] (2018). Small molecules to target the selenoprotein thioredoxin reductase. Chem-Asian J.

[bZhang2020] (2020). A novel thioredoxin-dependent peroxiredoxin (TPx-Q) plays an important role in defense against oxidative stress and is a possible drug target in *Babesia microti*. Front Vet Sci.

[bZhang2017] (2017). Targeting the thioredoxin system for cancer therapy. Trends Pharmacol Sci.

[bZhong2000] (2000). Structure and mechanism of mammalian thioredoxin reductase: the active site is a redox-active selenolthiol/selenenylsulfide formed from the conserved cysteine-selenocysteine sequence. Proc Natl Acad Sci USA.

